# Latest Developments and Future Perspectives in the Field Of Obesity

**DOI:** 10.17925/EE.2017.13.01.17

**Published:** 2017-04-03

**Authors:** Belén Pérez-Pevida, Alexander D Miras

**Affiliations:** Section of Investigative Medicine, Division of Diabetes, Endocrinology; Metabolism, Imperial College London, UK

**Keywords:** Obesity, cardiovascular outcomes, sodium-glucose co-transporter-2 inhibitors, glucagon-like peptide-1 agonists, endoscopic devices, bariatric surgery

## Abstract

The prevalence of obesity is increasing exponentially worldwide, becoming an international public health issue that affects quality of life, increases the risk of illness and raises healthcare costs in countries in all parts of the world. In this editorial, we analyse the latest progress in the management of obesity and associated cardiovascular risk factors, and summarise the latest randomised controlled trials that have had the biggest influence on the current changes we are experiencing in obesity management.

In the last five years, we have witnessed significant progress in the management of obesity and associated metabolic disorders, including type 2 diabetes mellitus (T2DM). The evidence comes from well-controlled randomised controlled trials (RCTs) and has changed the management of these conditions in clinic.

What we learned from the Action for Health in Diabetes (Look AHEAD) RCT is that an intensive weight loss intervention using numerous ‘tools’ including caloric restriction, meal replacement, increased physical activity and psychological support has impressive effects on the health of patients with T2DM.^1^ They not only lose 8.6% of their body weight, but also become healthier from a metabolic perspective and more functional. Disappointingly, this did not translate to reductions in cardiovascular mortality, unless the weight loss achieved was >10.0%. The Prevención con dieta Mediterránea (Prevention with Mediterranean diet, PREDIMED) RCT compared the effectiveness of a Mediterranean diet with extra virgin olive oil or nuts to a control diet – which was effectively a Western diet high in fat – in a Spanish population at high cardiovascular risk and a mean body mass index of 30 kg/m^2^.^2^ Interestingly, it demonstrated that patients on the Mediterranean diet lost negligible amounts of weight but had significant reductions in cardiovascular events compared to the control group. The message from these two trials is that whilst weight loss is always desirable, it does not necessarily translate to reductions in hard cardiovascular outcomes in the context of obesity or T2DM. In addition, it appears that the composition of the diet (i.e., high in monounsaturated fatty acids or polyunsaturated fatty acids) may have weight loss–independent effects on cardiovascular pathology.

In terms of pharmacotherapy for obesity and T2DM, we have learned that the use of anorexigenic gut hormone analogues (e.g. liraglutide) is effective not only for glycaemic control but also for moderate weight loss in the absence or presence of T2DM.^3,4^ These agents have a very acceptable safety profile, with no psychological or cognitive side effects. More importantly, in the context of T2DM and heart disease, they confer a survival benefit with a reduction in the rate of death from cardiovascular causes, nonfatal myocardial infarction or nonfatal stroke.^5–7^ These results come soon after the exciting results of the (Empagliflozin) Cardiovascular Outcome Event Trial in Type 2 Diabetes Mellitus Patients (EMPA-REG OUTCOME) trial, which demonstrated the sodium-glucose co-transporter-2 (SGLT-2) inhibitor empagliflozin, which blocks the reabsorption of glucose by the kidney, not only improves glycaemia, blood pressure and weight moderately, it also reduces the rates of death from cardiovascular causes by 38% in patients with T2DM and cardiovascular disease compared to placebo.^8,9^ We can probably infer that the glucagon-like peptide-1 (GLP-1) and SGLT-2 effects are class related and that their sister drugs are likely to demonstrate similar benefits. It would be intriguing to find out what effects the combination of these medications have on cardiovascular outcomes in patients with T2DM but also weight loss in obesity. There is also a move to use a combination of gut hormone analogues (e.g. GLP-1 with gastric inhibitory polypeptide or peptide YY), as the weight loss achieved may be additive or even synergistic.

Simultaneously, a number of centrally acting medications have been approved in some countries around the world. These include lorcaserin, the combination of topiramate with phentermine and the combination of naltrexone and bupropion.^10^ The combination therapies appear to be particularly effective in weight loss, but due to its mode of action, they unfortunately suffer from the almost inevitable psychological and neurocognitive side effects. They can therefore be used carefully in patients that are well monitored, and only continued longer term if the benefit:risk ratio is acceptable for the individual patient. The other conclusion that can be drawn from obesity medications, independent of their mode of action, is that their efficacy can be judged within the first three to four months of use. If they are not successful then, they should be discontinued, as they are unlikely to be of any benefit in the longer term. There is argument to use them in patients that have achieved weight stability, instead of weight gain, but the cost–benefit of such an approach is unlikely to be favourable.

In the field of medical devices, we have learnt that the duodenaljejunal bypass liner (EndoBarrier) induces significant weight loss in patients with and without T2DM through as yet unknown mechanisms.^11^ Disappointingly, the EndoBarrier Therapy for glycaemic improvement in inadequately controlled, obese, type 2 diabetic subjects on oral anti-diabetic agents (the ENDO trial) approval for the device was discontinued prematurely due to the higher than expected incidence of hepatic abscesses. It therefore remains to be seen what the future of this device will be and if these abscesses can be avoided. Another promising intervention, which is not actually a device, is endoscopic duodenal mucosa resurfacing, which involves the thermal ablation (or ‘burning’) of ~10 cm of the duodenal mucosa and results in moderate improvements in glycaemic control and a small amount of weight loss.^12^ The benefit of such an intervention is that no foreign body is left inside the gut. The mechanisms of its action remain unknown and intriguing. Another intervention that has raised eyebrows, but actually appears to work surprisingly well, is the AspireAssist device.^13^ This device consists of an endoscopically placed gastrostomy tube and siphon assembly, which allows patients to aspirate gastric contents, enabling them to remove approximately 30% of ingested calories. One would have expected that the ‘dumping’ of nutrients would result in rebound hunger and behavioural compensation, but there is no sign of this taking place. Implantable devices that alter vagal/neural signalling to the stomach have been tested in RCTs and even though they produced reasonable weight loss, this was not significantly different from the sham controls. This was probably because the dietary interventions in the control group were unexpectedly too effective.

Obesity surgery remains the ‘king’ of weight loss and metabolic interventions.^14^ Its clinical efficacy is supported by an increasing number of RCTs, two of them extending to five years of follow up. Surgery has an acceptable risk profile for many patients, but its use is still limited due to the high upfront costs but also from the – many times unsubstantiated – scepticism from healthcare professionals. The gastric bypass, vertical sleeve gastrectomy and gastric banding are the most popular procedures worldwide, with variations also increasing in popularity (e.g., the mini-gastric bypass). Hopefully surgery will become more acceptable to healthcare professionals and patients, and more widely offered in the cohort of patients where it is more cost effective.
